# Supplementary material for: A tuberculin skin test survey among Ghanaian school children
Source: BMC Public Health. 2010 Jan 26;10:35. doi: 10.1186/1471-2458-10-35 (PMC2829002; doi:10.1186/1471-2458-10-35)
Supplement: Additional file 1 — Tables S2 and S3. Table S2 - Prevalence of infection (with 95% confidence intervals) per district as estimated by different methods. Table S3 - Annual risk of tuberculosis infection (ARTI) (with 95% confidence intervals) per district as estimated by different methods. [file 1471-2458-10-35-S1.DOC]

**Table S2** Prevalence of infection (with 95% confidence intervals) per district as estimated by different methods

|  |  |  |  |  |  | **% with reaction size ≥ cut-off at** | | | | **Mirror method with mode at** | | | | |
| --- | --- | --- | --- | --- | --- | --- | --- | --- | --- | --- | --- | --- | --- | --- |
| **Region** | **District** | **n** | **% BCG** | **% non- reactor** | **Mean**  **age (yr)** | **10 mm** | **95%CI** | **15 mm** | **95%CI** | **16 mm** | **95% CI** | | **17 mm** | **95% CI** |
| Eastern | New Juaben | 3020 | 87.9 | 27.3 | 8.7 | **10.0** | (8.9-11.1) | **1.5** | (1.1-2.0) | **1.5** | (1.0-1.9) | | **1.0** | (0.7-1.4) |
|  |  |  |  |  |  |  |  |  |  |  |  | | |  |
| Central | AAK | 386 | 94.3 | 36.3 | 8.9 | **18.4** | (14.5-22.3) | **5.4** | (3.2-7.7) | **5.2** | (3.0-7.4) | **2.8** | | (1.2-4.5) |
|  | Cape Coast | 2784 | 92.9 | 33.7 | 8.8 | **12.8** | (11.6-14.1) | **2.5** | (1.9-3.1) | **2.2** | (1.6-2.7) | **1.4** | | (0.9-1.8) |
|  | KEEA | 604 | 81.0 | 16.2 | 8.7 | **12.6** | (9.9-15.2) | **4.6** | (3.0-6.3) | **4.6** | (3.0-6.3) | **2.6** | | (1.4-3.9) |
|  |  |  |  |  |  |  |  |  |  | |  |  | |  |
| Northern | SN | 622 | 91.8 | 56.8 | 8.6 | **3.7** | (2.2-5.2) | **0.5** | (0.0-1.0) | **0.2** | (0.0-0.5) | **0.0** | | (0.0-0.5) |
|  | Tamale | 1906 | 94.8 | 48.9 | 8.6 | **3.4** | (2.6-4.2) | **0.3** | (0.1-0.6) | **0.2** | (0.0-0.4) | **0.2** | | (0.0-0.3) |
|  | T-K | 652 | 94.2 | 56.1 | 8.4 | **3.4** | (2.0-4.8) | **0.0** | (0.0-0.5) | **0.0** | (0.0-0.5) | **0.0** | | (0.0-0.5) |
|  |  |  |  |  |  |  |  |  |  | |  |  | |  |
| Western | A-E | 2685 | 94.4 | 42.3 | 8.8 | **9.0** | (7.9-10.1) | **1.7** | (1.2-2.2) | **1.6** | (1.2-2.1) | **0.9** | | (0.6-1.3) |
|  | A-W | 572 | 94.4 | 56.1 | 9.3 | **12.2** | (9.6-14.9) | **3.7** | (2.1-5.2) | **3.7** | (2.1-5.2) | **2.4** | | (1.2-3.7) |
|  | W-W | 886 | 93.7 | 42.2 | 8.7 | **14.0** | (11.7-16.3) | **3.0** | (1.9-4.2) | **2.1** | (1.2-3.1) | **1.0** | | (0.4-1.7) |
|  |  |  |  |  |  |  |  |  |  | |  |  | |  |
| U-E | Bolga | 764 | 97.8 | 56.3 | 8.7 | **3.3** | (2.0-4.5) | **1.2** | (0.4-1.9) | **1.6** | (0.7-2.5) | **1.0** | | (0.3-1.8) |
|  |  |  |  |  |  |  |  |  |  | |  |  | |  |
|  |  |  |  |  |  |  |  |  |  | |  |  | |  |
| U-W | Wa | 613 | 97.7 | 51.4 | 8.9 | **4.4** | (2.8-6.0) | **0.8** | (0.1-1.5) | **0.3** | (0.0-0.8) | **0.0** | | (0.0-0.5) |
|  |  |  |  |  |  |  |  |  |  | |  |  | |  |
| Ashanti | AN | 235 | 60.9 | 67.7 | 8.8 | **5.1** | (2.3-7.9) | **1.7** | (0.0-3.4) | **0.9** | (0.0-2.0) | **0.9** | | (0.0-2.0) |
|  | BAK | 381 | 85.0 | 77.2 | 8.7 | **3.9** | (2.0-5.9) | **1.3** | (0.2-2.5) | **1.6** | (0.3-2.8) | **1.0** | | (0.0-2.1) |
|  | E-J | 130 | 63.8 | 56.9 | 8.5 | **10.0** | (4.8-15.2) | **3.8** | (0.5-7.2) | **5.4** | (1.5-9.3) | **3.8** | | (0.5-7.2) |
|  | Kumasi | 2342 | 77.8 | 52.6 | 8.6 | **11.1** | (9.8-12.4) | **2.9** | (2.2-3.5) | **2.6** | (2.0-3.3) | **1.7** | | (1.1-2.2) |
|  |  |  |  |  |  |  |  |  |  | |  |  | |  |
| G-A | Accra | 882 | 86.3 | 44.7 | 8.2 | **16.2** | (13.8-18.6) | **4.0** | (2.7-5.3) | **4.2** | (2.9-5.5) | **2.8** | | (1.7-3.9) |
|  | D-E | 165 | 97.0 | 18.2 | 8.7 | **18.8** | (12.8-24.7) | **4.2** | (1.2-7.3) | **3.6** | (0.8-6.5) | **1.8** | | (0.0-3.9) |
|  | D-W | 465 | 81.7 | 48.6 | 8.7 | **12.7** | (9.7-15.7) | **3.0** | (1.5-4.6) | **3.9** | (2.1-5.6) | **3.0** | | (1.5-4.6) |
|  | Ga | 811 | 78.3 | 44.1 | 8.7 | **14.7** | (12.2-17.1) | **4.2** | (2.8-5.6) | **4.1** | (2.7-5.4) | **2.5** | | (1.4-3.5) |
|  | Tema | 616 | 94.5 | 30.2 | 9.3 | **16.9** | (13.9-19.8) | **5.0** | (3.3-6.8) | **5.5** | (3.7-7.3) | **3.1** | | (1.7-4.4) |

AAK = Abura Asebu Kowmanse, KEEA = Komenda Edina Eguafo Abirem, SA = Savlegu Nanton, T-K = Tolon-Kumbungu, A-E = Ahanta-East, A-W = Ahanta-West, W-W = Wassa-West, U-E = Upper-East, U-W = Upper-West, AN = Atwima Nwobeagya, BAK = Bosumtwi Atwima Kwabianya, E-J = Ejisu-Juaben, G-A = Greater-Accra, D-E = Dangme-East, D-W = Dangme-West

**Table S3** Annual risk of tuberculosis infection (ARTI) (with 95% confidence intervals) per district as estimated by different methods

|  |  |  | **% with reaction size ≥ cut-off at** | | | | **Mirror method with mode at** | | | |
| --- | --- | --- | --- | --- | --- | --- | --- | --- | --- | --- |
| **Region** | **District** | **n** | **10mm** | **95%CI** | **15mm** | **95%CI** | **16mm** | **95%CI** | **17 mm** | **95%CI** |
| Eastern | New Juaben | 3020 | **1.2** | (0.8-1.6) | **0.2** | (0.0-0.3) | **0.2** | (0.0-0.3) | **0.1** | (0.0-0.2) |
|  |  |  |  |  |  |  |  |  |  |  |
| Central | AAK | 386 | **2.3** | (0.8-3.7) | **0.6** | (0.0-1.4) | **0.6** | (0.0-1.4) | **0.3** | (0.0-0.9) |
|  | Cape Coast | 2784 | **1.5** | (1.1-2.0) | **0.3** | (0.1-0.5) | **0.2** | (0.1-0.4) | **0.2** | (0.0-0.3) |
|  | KEEA | 604 | **1.5** | (0.6-2.5) | **0.5** | (0.0-1.1) | **0.5** | (0.0-1.1) | **0.3** | (0.0-0.8) |
|  |  |  |  |  |  |  |  |  |  |  |
| Northern | SA | 622 | **0.4** | (0.0-1.0) | **0.1** | (0.0-0.2) | **0.0** | (0.0-0.1) | **0.0** | -- |
|  | Tamale | 1906 | **0.4** | (0.1-0.7) | **0.0** | (0.0-0.1) | **0.0** | (0.0-0.1) | **0.0** | (0.0-0.1) |
|  | T-K | 652 | **0.4** | (0.0-0.9) | **0.0** | -- | **0.0** | -- | **0.0** | -- |
|  |  |  |  |  |  |  |  |  |  |  |
| Western | A-E | 2685 | **1.1** | (0.7-1.5) | **0.2** | (0.0-0.4) | **0.2** | (0.0-0.4) | **0.1** | (0.0-0.2) |
|  | A-W | 572 | **1.4** | (0.4-2.4) | **0.4** | (0.0-0.9) | **0.4** | (0.0-0.9) | **0.3** | (0.0-0.7) |
|  | W-W | 886 | **1.7** | (0.9-2.6) | **0.4** | (0.0-0.7) | **0.2** | (0.0-0.6) | **0.1** | (0.0-0.3) |
|  |  |  |  |  |  |  |  |  |  |  |
| U-E | Bolga | 764 | **0.4** | (0.0-0.8) | **0.1** | (0.0-0.4) | **0.2** | (0.0-0.5) | **0.1** | (0.0-0.4) |
|  |  |  |  |  |  |  |  |  |  |  |
| U-W | Wa | 613 | **0.5** | (0.0-1.1) | **0.1** | (0.0-0.3) | **0.0** | (0.0-0.2) | **0.0** | -- |
|  |  |  |  |  |  |  |  |  |  |  |
| Ashanti | AN | 235 | **0.6** | (0.0-1.6) | **0.2** | (0.0-0.8) | **0.1** | (0.0-0.5) | **0.1** | (0.0-0.5) |
|  |  |  |  |  |  |  |  |  |  |  |
|  | BAK | 381 | **0.5** | (0.0-1.1) | **0.2** | (0.0-0.5) | **0.2** | (0.0-0.6) | **0.1** | (0.0-0.5) |
|  | E-J | 130 | **1.2** | (0.0-3.1) | **0.5** | (0.0-1.6) | **0.6** | (0.0-2.0) | **0.5** | (0.0-1.6) |
|  | Kumasi | 2342 | **1.4** | 0.9-1.8 | **0.3** | (0.1-0.6) | **0.3** | (0.1-0.5) | **0.2** | (0.0-0.4) |
|  |  |  |  |  |  |  |  |  |  |  |
| G-A | Accra | 882 | **2.1** | (1.2-3.1) | **0.5** | (0.0-1.0) | **0.5** | (0.0-1.0) | **0.3** | (0.0-0.7) |
|  | D-E | 165 | **2.4** | (0.1-4.7) | **0.5** | (0.0-1.6) | **0.4** | (0.0-1.4) | **0.2** | (0.0-0.9) |
|  | D-W | 465 | **1.6** | (0.4-2.7) | **0.4** | (0.0-0.9) | **0.5** | (0.0-1.1) | **0.4** | (0.0-0.9) |
|  | Ga | 811 | **1.8** | (0.9-2.7) | **0.5** | (0.0-1.0) | **0.5** | (0.0-1.0) | **0.3** | (0.0-0.7) |
|  | Tema | 616 | **2.0** | (0.9-3.1) | **0.6** | (0.0-1.1) | **0.6** | (0.0-1.2) | **0.3** | (0.0-0.8) |

AAK = Abura Asebu Kowmanse, KEEA = Komenda Edina Eguafo Abirem, SA = Savlegu Nanton, T-K =Tolon-Kumbungu, A-E = Ahanta-East, A-W = Ahanta-West, W-W = Wassa-West, U-E = Upper-East, U-W =Upper-West, AN = Atwima Nwobeagya, BAK = Bosumtwi Atwima Kwabianya, E-J = Ejisu-Juaben, G-A =Greater-Accra, D-E = Dangme-East, D-W = Dangme-West
